# Thalamo-Sensorimotor Functional Connectivity Correlates with World Ranking of Olympic, Elite, and High Performance Athletes

**DOI:** 10.1155/2017/1473783

**Published:** 2017-02-02

**Authors:** Zirui Huang, Henry (Hap) Davis IV, Annemarie Wolff, Georg Northoff

**Affiliations:** ^1^Brain and Mind Research Institute, University of Ottawa, Ottawa, ON, Canada K1Z 7K4; ^2^Institute of Mental Health Research, University of Ottawa, Ottawa, ON, Canada K1Z 7K4; ^3^Swimming Canada, Calgary, AB, Canada T2P 3C5; ^4^Center for Cognition and Brain Disorders, Hangzhou Normal University, Hangzhou 311121, China; ^5^Zhejiang Key Laboratory for Research in Assessment of Cognitive Impairments, Hangzhou Normal University, Hangzhou 310015, China; ^6^Graduate Institute of Humanities in Medicine, Taipei Medical University, Taipei, Taiwan; ^7^Brain and Consciousness Research Center, Taipei Medical University-Shuang Ho Hospital, New Taipei City, Taiwan

## Abstract

Brain plasticity studies have shown functional reorganization in participants with outstanding motor expertise. Little is known about neural plasticity associated with exceptionally long motor training or of its predictive value for motor performance excellence. The present study utilised resting-state functional magnetic resonance imaging (rs-fMRI) in a unique sample of world-class athletes: Olympic, elite, and internationally ranked swimmers (*n* = 30). Their world ranking ranged from 1st to 250th: each had prepared for participation in the Olympic Games. Combining rs-fMRI graph-theoretical and seed-based functional connectivity analyses, it was discovered that the thalamus has its strongest connections with the sensorimotor network in elite swimmers with the highest world rankings (career best rank: 1–35). Strikingly, thalamo-sensorimotor functional connections were highly correlated with the swimmers' motor performance excellence, that is, accounting for 41% of the individual variance in best world ranking. Our findings shed light on neural correlates of long-term athletic performance involving thalamo-sensorimotor functional circuits.

## 1. Introduction

The human brain is remarkably malleable. This plasticity has been shown in musicians [1–4] and elite athletes who have undergone extensive motor training, such as Formula One racing, golf, badminton, and swimming [5–11]. The development of motor performance, especially at the expert level, results in systematic changes in the functional architecture and cortical recruitment in the human brain. Advances in functional magnetic resonance imaging (fMRI) have enabled us to characterize the development of brain systems in terms of functional networks [12, 13]. These networks are measured across the brain through resting-state functional connectivity (FC) [14, 15].

Despite evidence that training on motor tasks and related skill acquisition are associated with dynamic changes in resting-state FC [5, 6, 16–19], little is known about changes in functional circuits at exceptionally high levels of motor training, performance, and achievement. Specifically, the functional circuits' association with performance excellence, measured by world ranking, is yet to be elucidated.

**Figure 1:**
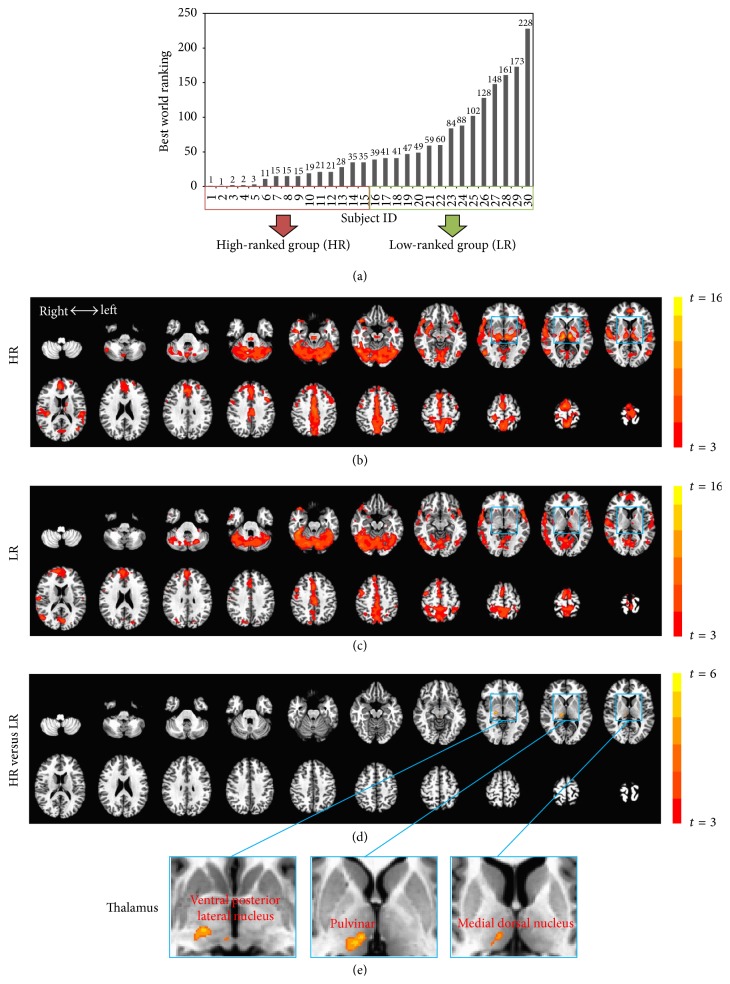
Whole-brain functional connectivity (FC). (a) High-ranked (HR; *n* = 15, rank: 1–35) and low-ranked (LR; *n* = 15, rank: 39–228) swimmers were divided by a median split in world rankings. (b) The number of functional connections (positive correlations) between an individual voxel and all remaining voxels in the brain was computed by degree of centrality analysis. Whole-brain FC map for HR swimmers is shown by a one-sample *t*-test (against zero). (c) Whole-brain FC map for LR swimmers. (d) HR swimmers show stronger net connections between the right thalamus and the whole brain when compared to those in the LR by a whole-brain two-sample *t*-test. (e) Magnified views of the thalamus show significant group differences in the ventral posterior lateral nucleus, the pulvinar, and the medial dorsal nucleus. All resulting *t*-maps were thresholded at a corrected *p* value of <0.05.

Using resting-state fMRI, we studied a unique sample of world-class athletes: 30 Olympic and elite internationally ranked swimmers with career-highest world ranking from 1st to a cutoff at 250 (Figure 1(a)). Each participant had prepared for participation in the Olympic Games. All participants were calculated to have trained for a minimum of 15 years (training in the last five years was conservatively estimated at 15,000 hours) before fMRI scanning. To our knowledge, no study has yet been conducted among the most elite with such long training histories.

Initially, the method employed to address the aforementioned research question was a data-driven, graph-theoretical FC approach, including a whole-brain degree of centrality (DC) calculation [20–23]. The aim was to identify brain regions with brain-wide net connections which differentiate high-ranked (HR) and low-ranked (LR) participants. Next, we sought to characterize the pathway of differing FC between HR and LR participants. This was achieved using seed-based FC analysis [14, 24]. Finally, correlation analyses between FC values and best world ranking of the athletes were performed. Our hypothesis was that athletes who had engaged in long-term and demanding athletic training would show strengthened functional connections in sensorimotor-related functional circuits, in correlation with demonstrated performance excellence. Confounding factors such as years of training (practice years), age, and the time of entry into the study were accounted for.

## 2. Methods

### 2.1. Participants

The sample of 30 swimmer participants included Olympic Games and World Championships competitors together with their nonelite though internationally ranked cohorts (16 female, age: 18–29; 14 male, age: 18–29). The best world ranking ranged from 1st to a cutoff at 250 (mean overall: 56). For the purpose of forming a meaningful contrast to the elite swimmers, participants were divided by median split into two equal groups: high-ranked Olympic and World Championships competitors (HR; *n* = 15, rank: 1–35) and low-ranked individuals (LR; *n* = 15, rank: 39–228). None in the LR group had competed in Olympic Games or World Championships competitions. The comparison group of low-ranked swimmers with world ranking of 39–228 were labeled “nonelite” because none in this group had ever competed in major international games. Thus, the elite swimmers, world-ranked 1–35, were internationally competitive and were contrasted to swimmers who had trained to compete at the highest levels but never qualified to do so. World ranking was deemed the superior measure of acquired motor skill; among the world's best athletes, both team selection and podium achievement are founded to be the basis of ranking, not time. Practice years of specific motor training were defined as the period from year of a swimmer's first national qualification (a standard marking the athlete's eligibility to compete for a position on a national team) to the year of highest or best world ranking. The year of best world ranking is defined as that year in which the athlete's fastest competitive time for all competitions within a 12-month period achieved the highest ranking among all international competition times for that same period. All athletes in the sample are estimated to have engaged in specific motor training as competitive swimmers for at least 15 years, with a conservative estimate of well over 15,000 hours in the immediate 5 years before fMRI scanning. Participants had no history of major psychiatric or neurological disorders and were medication-free at the time of scanning. Written informed consent was obtained from each subject before the experiment. This study was approved by the ethics committees at both University of Ottawa and University of British Columbia. The methods were carried out in accordance with the approved guidelines and regulations.

### 2.2. fMRI Data Acquisition

A Philips Achieva 3.0T scanner with a standard head coil was used to acquire gradient-echo EPI images of the whole brain (TR, 1.0 s; TE, 30 ms; 21 slices; slice thickness = 6 mm; spacing = 0; field of view = 210 mm; flip angle = 76°; image matrix: 64 × 64; voxel size: 3.28 × 3.28 × 6.00 mm^3^; number of fMRI frames: 360). During the 6-minute resting-state fMRI scan, subjects were instructed to relax, stay awake, and have their eyes closed; postscan self-report questionnaires indicated that subjects did comply with these instructions. Time-locked cardiac and respiratory signals were recorded. High-resolution anatomical images were acquired at the end of the experiment. There was no other task in the scanner prior to resting-state data acquisition.

### 2.3. fMRI Data Preprocessing

Preprocessing steps were implemented in AFNI (http://afni.nimh.nih.gov/afni). Firstly, physiological noise correction consisted of removal of time-locked cardiac and respiratory artifacts using RETROICOR [25, 26]. Next, the functional images from each scan were aligned (head motion correction), slice timing corrected, temporally standardized, resampled to 3 × 3 × 3 mm^3^, spatially smoothed (6 mm full width at half maximum Gaussian blur), and transformed into Talairach space, and linear trends were removed. The data was then filtered with a band-pass filter preserving signals between 0.01 and 0.1 Hz which is thought to reflect fluctuations of spontaneous brain activity [14]. The estimated six parameters of head motion and mean time series from the white matter (WM) and cerebrospinal fluid (CSF) were regressed out. To minimize partial voluming with gray matter, the WM and CSF masks were eroded by one voxel [27].

The issue of motion artifacts was addressed rigorously as minor group differences in motion have been shown to artificially create between-groups differences [28, 29]. We first confirmed that the maximal head motion of all participants was less than one voxel size (3 mm in shift or 3° in rotation). Next, we calculated the indices of the amount of motion for each subject [30]. No group difference (high-ranked versus low-ranked) was observed for either shift (*t* = 0.17; *p* = 0.87) or rotation (*t* = 0.015; *p* = 0.99) by two-sample *t*-tests. To further exclude the potential confoundedness of head motion, we performed standard GLM analyses, including head motion indices as covariates, for all the following group-level analysis.

### 2.4. Degree of Centrality

Using a data-driven approach, we performed whole-brain voxel-based degree of centrality (DC) analysis. In graph theory, a complex system is modeled as a “graph,” which is defined as a set of “nodes” linked by “edges.” For a binary graph, DC is the number of edges connecting to a node. For a weighted graph, DC is defined as the sum of weights from edges connecting to a node, which is also sometimes referred to as the node strength [22, 23]. The DC analysis was performed for each subject by AFNI program* 3dTcorrMap*. Specifically, voxel-based graphs were generated for each participant. Each voxel constitutes a node in the graph and each significant functional connection (Pearson's correlation) between any pair of voxels is an edge. A voxel-based graph is thus a mathematical representation of the functional network consisting of nodes or voxels and their edges or connections [20–22]. To obtain each participant's graph, the correlation between the time series of each voxel and every other voxel in the individual whole-brain mask was computed. A binary, undirected adjacency matrix was then obtained by thresholding each correlation at *r* > 0.3 [22]. Based on the graph, DC was calculated at the individual level. We computed DC by counting the number of functional connections (positive correlations) between each voxel and all other voxels. Finally, normalized DC indices were calculated by transforming DC to *Z*-scores based on the global mean of DC and standard deviation across voxels in the brain.

Group-level analysis of DC was performed by contrasting HR versus LR by two-sample *t*-test with head motion matrices, age, and gender as covariates. One-sample *t*-test (against zero) was also performed for each group. Unless otherwise stated, all resulting *t*-maps were thresholded at a corrected *p* value of <0.05. That is, the multiple comparison error was corrected using Monte Carlo simulation as implemented in AFNI program* AlphaSim*, yielding a family-wise error rate (FWER) at *p* < 0.05. The smoothness used in* AlphaSim* was the average smoothness across subjects.

### 2.5. Functional Connectivity

To characterize the pathway of differing functional connection between HR and LR participants, we performed a seed-based functional connectivity (FC) analysis, using a seed region (DC-seed) defined by the group contrast of DC. A voxel-wise FC map to the seed was computed as a map of temporal correlation coefficients between blood oxygenation level dependent (BOLD) time course in each voxel and BOLD time course averaged across voxels in the seed region [14, 24]. FC maps were created for each subject and then transformed by Fisher's *Z* transform for second-level one-sample and two-sample *t*-tests. Group-level analysis of FC was performed in a similar way as that of DC.

### 2.6. Confirmation by Region of Interest (ROI) Analysis

To identify the sensorimotor network (SMN) of all swimmers, FC with a seed region in the left central sulcus was used. The seed region was voxels in a sphere of 6 mm radius centred on stereotactic coordinates reported by a previously published focus (Talairach coordinates: [−36, −24, 49] [31]). FC maps were created for all subjects and then transformed into Fisher's *Z* for second-level one-sample *t*-tests (*n* = 30). The group FC map of the somatosensory network was defined as voxels at threshold *p* < 0.001 (corrected, cluster extent >100 voxels) [32–34]. Nodes of somatosensory network were defined as voxels in a sphere of 6 mm radius centred on each cluster of the networks. The largest 3 clusters, sorted by cluster size, in the somatosensory network were defined as ROIs in the subsequent analysis. For each subject, the averaged FC value across voxels within a given ROI was extracted from the individual FC map of DC-seed (see above). Of note, the FC value of a given ROI quantified the strength of connection between this ROI and the DC-seed. Finally, two-sample *t*-tests (HR versus LR) of FC values were performed for all ROIs, respectively.

### 2.7. Intersubject Correlation Analysis

To investigate the predictive value of FCs for performance excellence, we associated the individual FC value of each ROI with best world ranking. Spearman's correlation analyses were performed across the 30 subjects with a 95% confidence interval based on 1000 bootstrap samples. To ensure that the correlations between the FCs and best world ranking were not confounded by practice years, age, or the time of entry into the study, we performed partial correlation analyses (95% confidence interval; 1000 bootstrap samples) between the FCs and best world ranking by including these factors as covariates, respectively.

## 3. Results

### 3.1. Higher Thalamus DC in the High-Ranked Group

The numbers of functional connections (positive correlations) between an individual voxel and all remaining voxels in the brain were compared via DC analysis. HR and LR swimmers shared a similar pattern of cortical connections, yet HR swimmers appeared to have stronger cortical connections in the thalamus (Figures 1(b) and 1(c)). This was examined by a whole-brain two-sample *t*-test contrasting HR versus LR. Only one significant cluster (67 voxels; Talairach coordinates of best voxel: [11, −25, 2]) was found which included the ventral posterior lateral nucleus, the pulvinar, and the medial dorsal nucleus, located in the right thalamus; HR swimmers showed stronger net connections between the thalamus and the whole brain when compared to those in the LR (Figures 1(d) and 1(e)).

### 3.2. Stronger FC of Thalamo-Sensorimotor Circuits in the High-Ranked Group

**Figure 2:**
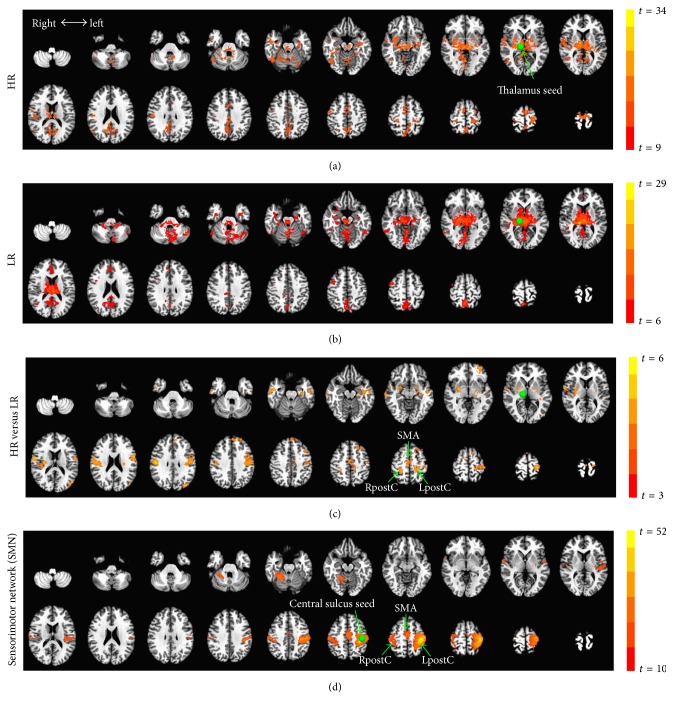
Seed-based functional connectivity (FC). (a) Thalamus-seed FC map for high-ranked (HR) swimmers is shown by a one-sample *t*-test (against zero) with a threshold of top 10% voxels of the whole brain (*p* = 1.6*e* − 7). (b) Thalamus-seed FC map for low-ranked (LR) swimmers (top 10% voxels; *p* = 3.2*e* − 5). (c) Contrast map for HR versus LR by a two-sample *t*-test (*p* < 0.05, corrected). (d) The sensorimotor network (SMN), including the supplementary motor area (SMA) and both the right postcentral gyrus (RpostC) and left postcentral gyrus (LpostC), is identified by left central sulcus-seed FC (top 5% voxels; *p* = 8.0*e* − 14) in the entire group (*n* = 30).

To characterize the pathway which locates a stronger connection to the thalamus in the HR group, a seed-based FC analysis, using the above thalamus cluster as a seed region, was performed. The HR swimmers showed stronger functional connectivity between the thalamus and regions particularly in the sensorimotor network (SMN), including the supplementary motor area (SMA), right postcentral gyrus (RpostC), and left postcentral gyrus (LpostC) (Figures 2(a), 2(b), and 2(c)).

**Figure 3:**
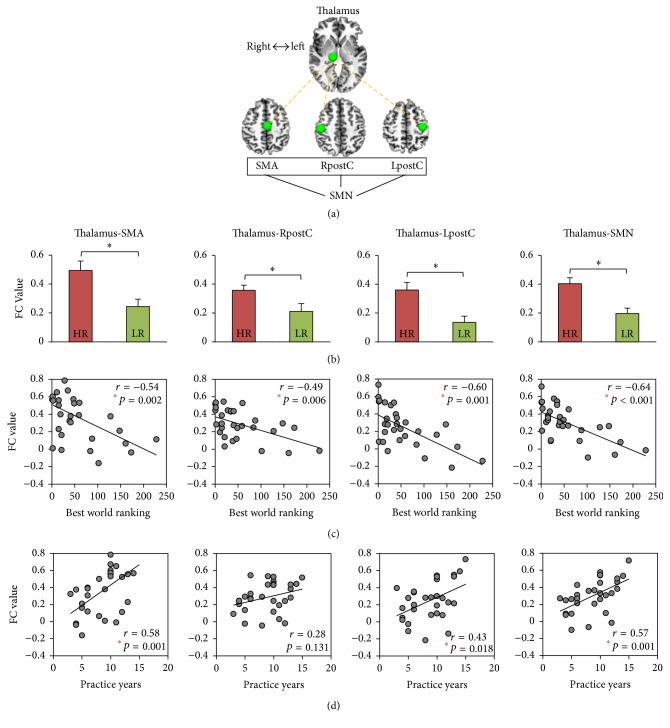
Functional connectivity (FC) of thalamo-sensorimotor circuits and its associations with performance excellence (best world ranking) and motor training (practice years). (a) An illustration of the regions of interest (ROIs) within the thalamo-sensorimotor circuits. The right thalamus is defined by the group difference in the degree of centrality analysis. The sensorimotor network (SMN), including supplementary motor area (SMA) and both the right postcentral gyrus (RpostC) and left postcentral gyrus (LpostC), is defined by left central sulcus-seed FC. (b) Group comparison for high-ranked (HR) versus low-ranked (LR) swimmers in the thalamo-sensorimotor FCs (^*∗*^*p* < 0.05; error bars indicate ±SEM). (c) Correlations between the thalamo-sensorimotor FCs and best world ranking (*n* = 30). (d) Correlations between the thalamo-sensorimotor FCs and practice years (*n* = 30). Spearman's correlation analysis with a 95% confidence interval based on 1000 bootstrap samples was used.

We confirmed that these regions do indeed pertain to the SMN by an independent FC analysis in the entire group (*n* = 30), using the left central sulcus (Talairach coordinates: [−36, −24, 49] [31]) as a seed region (Figure 2(d)). On this basis, we defined the SMA, RpostC, and LpostC as independent ROIs and avoided any double-dipping on the data. This also helps verify the whole-brain analysis and quantify the strength of FC between these ROIs and the thalamus (Figure 3(a)). As expected, stronger FC of thalamus-SMA (*p* = 0.013), thalamus-RpostC (*p* = 0.018), and thalamus-LpostC (*p* = 0.006) was observed when the HR was compared to the LR. The average of the three, thalamus-SMN (*p* = 0.002), was also found to have higher FC in the HR group (Figure 3(b)).

### 3.3. Thalamo-Sensorimotor FC Correlates with Performance Excellence

Exploring the predictive value of FCs for performance excellence, we associated the individual FC values between the thalamus and regions in the SMN with the best world ranking across the 30 subjects. Interestingly, we found significant correlations between all pairs of FCs and the best world ranking: rank *∝* thalamus-SMA (*r* = −0.54; *p* = 0.002); rank *∝* thalamus-RpostC (*r* = −0.49; *p* = 0.006); rank *∝* thalamus-LpostC (*r* = −0.60; *p* = 0.001); rank *∝* thalamus-SMN (*r* = −0.64; *p* < 0.001) (Figure 3(c)). The significance for the above correlations was significant enough to survive a Bonferroni correction for multiple comparisons. Of note, the highest correlation with world ranking was the FC between the thalamus and SMN (*r*^2^ = 0.41). Here, the FC of thalamus-SMN accounted for 41% of the total variance of best world ranking in the swimmers.

We explored the following possible confounding variables to our results: years of training (practice years), age, and the time of entry into the study. (1) It was considered that the correlation between the FCs and best world ranking may be confounded by practice years as years had correlated significantly both with world ranking (*r* = −0.54; *p* = 0.002) and with FCs (Figure 3(d)). To address this, we examined the correlations between the FCs and best world ranking using partial correlation analyses, including practice years as a covariate. All correlations remained significant: rank *∝* thalamus-SMA (*r* = −0.40; *p* = 0.032); rank *∝* thalamus-RpostC (*r* = −0.47; *p* = 0.011); rank *∝* thalamus-LpostC (*r* = −0.56; *p* = 0.002); rank *∝* thalamus-SMN (*r* = −0.57; *p* = 0.001). (2) Next, we examined age as a possible confounding variable [35]. This possibility was also ruled out in our data. Results remained significant after regressing out age: rank *∝* thalamus-SMA (*r* = −0.43; *p* = 0.020); rank *∝* thalamus-RpostC (*r* = −0.48; *p* = 0.009); rank *∝* thalamus-LpostC (*r* = −0.57; *p* = 0.001); rank *∝* thalamus-SMN (*r* = −0.59; *p* = 0.001). (3) Finally, we assessed whether the time of entry into the study might affect our main findings with years from best ranking to fMRI scanning potentially showing group differences. All FCs correlations with best world ranking remained significant after regressing out the years from best ranking to fMRI scanning: rank *∝* thalamus-SMA (*r* = −0.43; *p* = 0.019); rank *∝* thalamus-RpostC (*r* = −0.49; *p* = 0.007); rank *∝* thalamus-LpostC (*r* = −0.57; *p* = 0.001); rank *∝* thalamus-SMN (*r* = −0.59; *p* = 0.001). In short, the results suggested that a strong FC between the thalamus and SMN regions associates with higher world ranking, irrespective of years of training, age, and the time of entry into the study.

**Figure 4:**
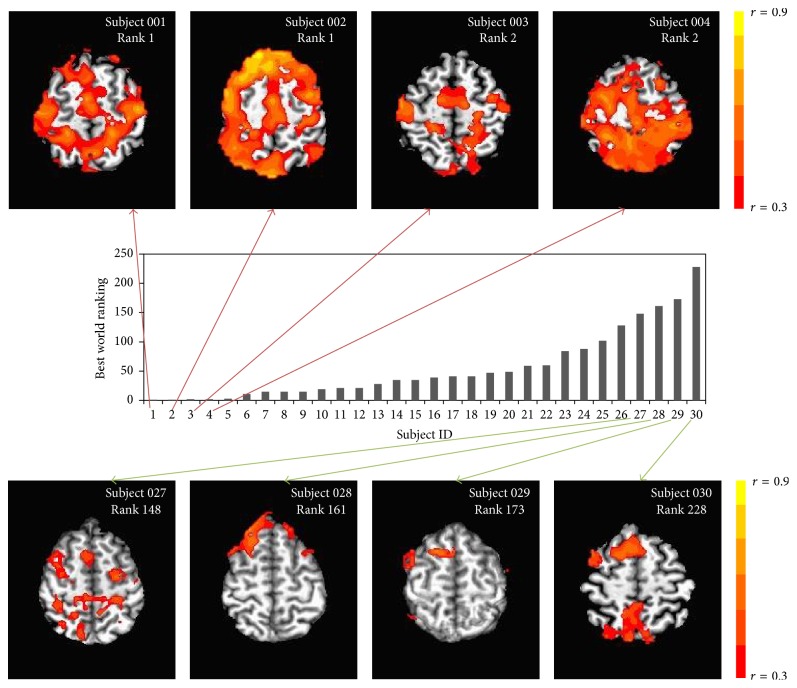
Thalamus-seed functional connectivity maps for eight representative individuals thresholding at voxel-wise correlation coefficient (*r*) > 0.3.

To illustrate this relationship between performance excellence (world ranking) and FC, we depict (as a representation only) a contrast of the top four from our elite sample (persons whose personal best merited a career-best world ranking of 1st or 2nd) with a selection of four from among those with best world ranking that was well below this (Figure 4). While future study may explore a contrast of distance and sprint swimmers (specialists at 1500 m as compared with 100 m), we note that both were represented in the subsample of the top four subjects.

## 4. Discussion

We have studied functional connectivity in elite athletes with multiyear training history and exceptional international standing. The sample size, level of achievement, and long-term training history make this sample of elite athletes an especially unique and important contribution to the literature. We found that the right posterior thalamus has strongest connections to the sensorimotor network in more elite swimmers, those with the highest best world ranking (career-best rank: 1−35). Importantly, these functional connections were highly correlated with the swimmers' best world ranking.

While motor-related cortices, primary motor cortex, supplementary motor areas, and cerebellum, are often suggested to be critical to motor training [36–40], these findings may overlook the functional significance of a central gateway of our brain, the thalamus, signified by its key function on relaying neural signals from nigra, globus pallidus, and cerebellum motor signals to the cerebral cortex [41–44]. The relaying functions give the thalamus a crucial role in mediating motivation, planning, and goal-directed behaviors [45].

Our data-driven, unbiased approach led us to identify the thalamus and suggested an intriguing possibility: the thalamo-sensorimotor functional circuits mediate the superior motor skills and performance excellence in elite athletes. We note that these functional circuits included several subregions in the thalamus, such as the ventral posterior lateral nucleus, the pulvinar, and the medial dorsal nucleus. These subregions are thought to play important roles in multisensory and sensorimotor integrations, for example, integrating different sensory modalities with motor attributes [46]. We therefore suggest that these integrative processes are essential for performance excellence.

The major difference between high-ranked and low-ranked swimmers was only found in the right thalamus. This may appear counter-intuitive, as swimming uses both sides of the body; one might thus expect bilateral thalamus changes. We consider it possible that our limited sample size accounts for statistical significance for only the right thalamus, with the left thalamus not reaching significance after multiple comparison corrections. This interpretation is supported by the one-sample *t*-test results in Figures 1(b) and 1(c) showing stronger bilateral thalamus-cortical connections in the HR group alone. Therefore, based on these observations, we would not suggest hemisphere dominance in terms of the thalamo-sensorimotor functional circuits.

A swimmer's achievement of an elite world ranking is, presumably, a function of multiple interactive factors including training and competitive history, social relationships, body geometry, and motor coordination, together with cognitive, affective, and motivational factors. Acting in concert, these promote effective training outcomes; we cannot yet specify what is responsible for the observed FC group differences. Thus, future studies, among highly elite individuals who have engaged in long-term training, must seek to disentangle and reveal those elements best related to plastic changes of thalamo-sensorimotor functional circuits.

It is also worth pointing out that we chose to use the swimmers' world ranking rather than their elapsed time in an event as a measure of performance excellence. The reasons were the following: (i) a swimmer's time at Olympic Games may be different from his or her time at subsequent World Championships months later. What matters most to a world-class athlete, and to the country he or she represents, is the achieved ranking. Sponsorships, monetary rewards, and other incentives apply after a ranking is achieved for a posted time and not after a swimmer posts his or her best time. If the swimmer's time improves but the times of their competitors improve more, then the swimmer's ranking will drop; rewards are distributed more reliably to a ranking than to a fast time. Time is not determinative as to whether the athlete achieves accolades for a win; (ii) the swimmers obtained the ranking in across different swimming strokes and distances, each with an influence on time. Sorting the swimmers using time would not enable between-swimmer comparisons on performance.

Finally, future research may also explore whether the functional plasticity of the presented thalamo-sensorimotor circuits accompanies structural changes such as white matter fiber density. Longitudinal study combined with anatomical/structural investigation using diffusion tensor imaging (DTI) has the potential to further extend our findings.

## 5. Conclusions

A unique sample of especially accomplished, highly trained athletes from long-term athletic training programs showed strengthened thalamo-sensorimotor functional circuits. Importantly, the strength of FC was remarkably correlated with performance excellence. Our findings hold important implications as a possible thalamo-sensorimotor “functional marker,” which may become a neural predictor of world ranking in elite athletes. If validated, this may even lead to research isolating training-specific elements associated with strengthened thalamocortical functional connections.
